# Mechanism of negative modulation of FSH signaling by salt-inducible kinases in rat granulosa cells

**DOI:** 10.3389/fendo.2022.1026358

**Published:** 2022-09-29

**Authors:** Marah Armouti, Miriam Rodriguez-Esquivel, Carlos Stocco

**Affiliations:** Department of Physiology and Biophysics, University of Illinois at Chicago, Chicago, IL, United States

**Keywords:** ovary, granulosa cells, SIKs, CREB, CRTC2, steroidogenesis

## Abstract

The optimal development of preovulatory follicles needs follicle-stimulating hormone (FSH). Recent findings revealed that salt-inducible kinases (SIKs) inhibit FSH actions in humans and rodents. This report seeks to increase our understanding of the molecular mechanisms controlled by SIKs that participate in the inhibition of FSH actions in primary rat granulosa cells (GCs). The results showed that FSH causes a transient induction of *Sik1* mRNA. In contrast, SIK inhibition had no effects on FSH receptor expression. Next, we determined whether SIK inhibition enhances the effect of several sequential direct activators of the FSH signaling pathway. The findings revealed that SIK inhibition stimulates the induction of steroidogenic genes by forskolin, cAMP, protein kinase A (PKA), and cAMP-response element-binding protein (CREB). Strikingly, FSH stimulation of CREB and AKT phosphorylation was not affected by SIK inhibition. Therefore, we analyzed the expression and activation of putative CREB cofactors and demonstrated that GCs express CREB-regulated transcriptional coactivators (CRTC2) and that FSH treatment and SIK inhibition increase the nuclear expression of this factor. We concluded that SIKs target the FSH pathway by affecting factors located between cAMP/PKA and CREB and propose that SIKs control the activity of CRTC2 in ovarian GCs. The findings demonstrate for the first time that SIKs blunt the response of GCs to FSH, cAMP, PKA, and CREB, providing further evidence for a crucial role for SIKs in regulating ovarian function and female fertility.

## Introduction

Ovulation is the pinnacle of folliculogenesis, a process requiring granulosa cell (GC) proliferation and differentiation, both needed for preovulatory follicle formation. The optimal development of preovulatory follicles needs follicle-stimulating hormone (FSH). We recently revealed novel roles for a family of kinases named salt-inducible kinase (SIKs) in regulating folliculogenesis and ovulation in rodents and controlling GC differentiation in humans and rodents (1). In particular, we demonstrated that SIK2 blunts FSH-induced GC differentiation and restricts the number of follicles reaching ovulation. Thus, our previous studies demonstrated that inhibition of SIK activity *in vivo* or *in vitro* potentiates the stimulatory effect of FSH on GC differentiation and estradiol production. Therefore, SIKs are key regulators of the response of GCs to gonadotropins and consequently control ovulation efficiency and fertility. These results motivate studies aimed at determining the specific signaling mechanisms targeted by SIKs in GCs.

The three SIK isoforms (SIK1, SIK2, and SIK3) are serine/threonine protein kinases of the AMP-activated kinases family known to regulate metabolism, cancer, melanocytes, and bone formation (2, 3). SIK1 was identified in the adrenal gland of rats fed a high-salt diet (4, 5). Nevertheless, SIK1 is also induced by glucagon in β-cells (6) and depolarization in neurons (7). In contrast, SIK2 and SIK3 are expressed ubiquitously, with the highest levels in the adipose tissue for SIK2 and the brain for SIK3. SIKs have an N-terminal kinase domain, a central ubiquitin (UBA) domain, and a C-terminal containing potential PKA phosphorylation sites (8). The function of the C-terminal region and the UBA domain remains unknown. In contrast, it is known that SIK activity depends on the phosphorylation of Thr residues in the N-terminal kinase domain by liver kinase 1 (LKB1, also known as serine/threonine kinase 11) (9). We have shown that in human and rodent GCs, SIK2 and SIK3 are the most prominent isoforms (1). However, the mechanisms controlling SIKs expression and activity in GCs have not been explored.

In GCs, the FSH receptor primarily activates Gα protein, which in turn stimulates adenylate cyclase (AC) activity and the production of cyclic adenosine 3′,5′-monophosphate (cAMP). cAMP leads to the activation of protein kinase A (PKA) and cAMP response element-binding protein (CREB), leading to the induction of markers of GC differentiation, including *Cyp19a1* (also known as aromatase) (10–12), steroidogenic-acute regulator (*Stard1*, most commonly referred to as StAR) (13), and *Cyp11a1* (most commonly referred to as P450scc) (14). Here, we seek to increase our understanding of the molecular mechanisms controlled by SIKs involved in regulating these genes and investigate the effects of SIK inhibition at all levels of the FSH receptor signaling pathway. We also tested if SIKs affect the expression of the FSH receptor. In addition, we examined if FSH regulates the expression of SIKs isoforms in primary ovarian GCs. Our findings demonstrate that SIK activity regulates the nuclear localization of CREB coactivators downstream of PKA.

## Materials and methods

Cells – GCs were isolated from 23-25 days old estradiol-treated immature rats and cultured as described previously (15–17). The use of a GC culture system from estradiol-treated immature rats is a well-established and valuable approach that provides an *in vitro* model for examining GC differentiation and the mechanisms involved in the regulation of GCs by FSH (18). Cells were treated with ovine FSH, forskolin, db8CPT, or dbcAMP with or without HG-9-91-01 (HG), a specific inhibitor of SIKs. All inhibitors and hormones were obtained from Tocris (Bristol, United Kingdom). The Institutional Animal Care and Use Committee at the University of Illinois at Chicago approved all animal experiments.

RNA isolation and quantification – Total RNA was isolated using TRIzol (Invitrogen, Carlsbad, CA) and reverse-transcribed using anchored oligo-dT primers (IDT, Coralville, IA) and Moloney Murine Leukemia Virus reverse transcriptase (Invitrogen). Intron-spanning primers were used to amplify the gene of interest (GOI) along with a standard curve containing serial dilutions of the cDNA of the GOI. Real-time PCR amplifications were performed with Brilliant II qPCR SYBR master mix (Agilent, Santa Clara, CA) using an AriaMx instrument (Agilent). For each sample, the number of cDNA copies corresponding to 10 ng of total RNA was computed for each GOI and ribosomal protein L19 (*Rpl19*). Then, the expression of each GOI is reported as the ratio between the number of copies of the GOI and *Rpl19*.

Promoter Reporter Assays - The CRE-Luc reporter was generated by cloning three copies of the cAMP response element (TGACGTCA) followed by the firefly luciferase cDNA (CRE-Luc). Lentiviruses containing this construct were generated using 293FT cells (Invitrogen) as previously described (19). Cells were infected with lentiviruses and, after overnight incubation, treated as indicated in the figure legends. Empty plasmids were used as controls. Luciferase activity was determined in 50 μl of lysates and expressed relative to renilla luciferase, as previously described (19).

Overexpression experiments – Expression plasmids encoding constitutively active PKA (20) or C2/CREB (21) were kindly provided by Dr. Anthony J. Zeleznik (University of Pittsburgh) and Dr. Thiel (University of Saarland, Germany), respectively. C2/CREB cDNA was subcloned into the pGPcs vector, which was derived from the pCDH vector (System Biosciences, Mountain View, CA). The capacity of these plasmids to activate the CRE-Luc reporter was tested by transfecting the CRE-Luc along with pGPcs, caPKA, or C2/CREB in HEK293 cells using calcium phosphate precipitation (**Supplemental Figure 1**). CREB-regulated transcriptional coactivators (CRTCs) contain a highly conserved N terminal CREB-binding domain (CBD) that is responsible for interacting with the transcription factor CREB (22). We generated a lentiviral pGPcs-based dominant negative CRTC (CRTC-DN) construct that expresses only the CBD (1–54 aa) of mouse CRTC2. CRTC-DN is predicted to bind CREB but lacks the transcriptional activation domain, consequently interfering with the functions of endogenous CRTCs through competitive CREB binding (23, 24). Lentivirus stocks were generated in HEK293 cells (Invitrogen) transfected with pGPcs (empty), caPKA, C2/CREB, or CRTC2-DN lentiviral vector along with the packaging and envelope plasmids psPAX2 and pMD2G (Addgene, Watertown, MA). Cell supernatants were concentrated by ultracentrifugation. Viral stocks were titrated in 293FT cells aided by a fluorescence marker. Viral stocks carrying pGPcs (control), caPKA, C2/CREB, or CRTC*-*DN were added directly to the cells 2 h after plating at a multiplicity of infection of 20 and cultured for 24 hours before the initiation of the treatments described in each figure.

Western blot analysis *-* Cytosolic and nuclear extracts were prepared as described previously (25). Protein concentration was determined using Pierce BCA Protein Assay Kit (Thermo Fisher Scientific, Rockford, Illinois). Proteins were subjected to gel electrophoresis, transferred to nitrocellulose membranes, and processed by routine procedures. The primary antibodies and the dilutions used were Lamin B1 (1:500), GAPDH (1:500), CRTC1 (1:1000), CRTC2 (1:1000), CRTC3 (1:1000), AKT (1:1000), S473-AKT (1:1000), CREB (1:1000), and S133-CREB (1:1000), all from Cell Signaling (Danvers, MA). The secondary antibodies used were anti-rabbit IgG-HRP (goat, 1:10,000) from Abcam (Cambridge, United Kingdom) or anti-mouse IgG-HRP (goat, 1:10,000) from Jackson ImmunoResearch Laboratory Inc. (West Grove, PA). Detection was performed with Supersignal West Femto Maximum Sensitivity Substrate (Thermo Scientific, Rockford, IL) and detected using ChemiDoc MP Imaging System (BioRad, Hercules, CA). Protein expression quantification was performed with ImageJ software (National Institutes of Health, Bethesda, Maryland).

Statistics – All experiments were repeated three times or more as indicated in the figure legend. Determinations of mRNA levels or luciferase activity were run in duplicate. Data were analyzed using Prism 6 (San Diego, CA). Differences between two groups were determined by Student’s *t*-test. For multiple groups, one-way ANOVA was used, and differences between individual means were determined by the Tukey test. Data from all experiments are plotted as mean ± SEM. Significant differences were recognized at *p* < 0.05.

## Results

### Effects of FSH on SIKs expression

**Figure 1:**
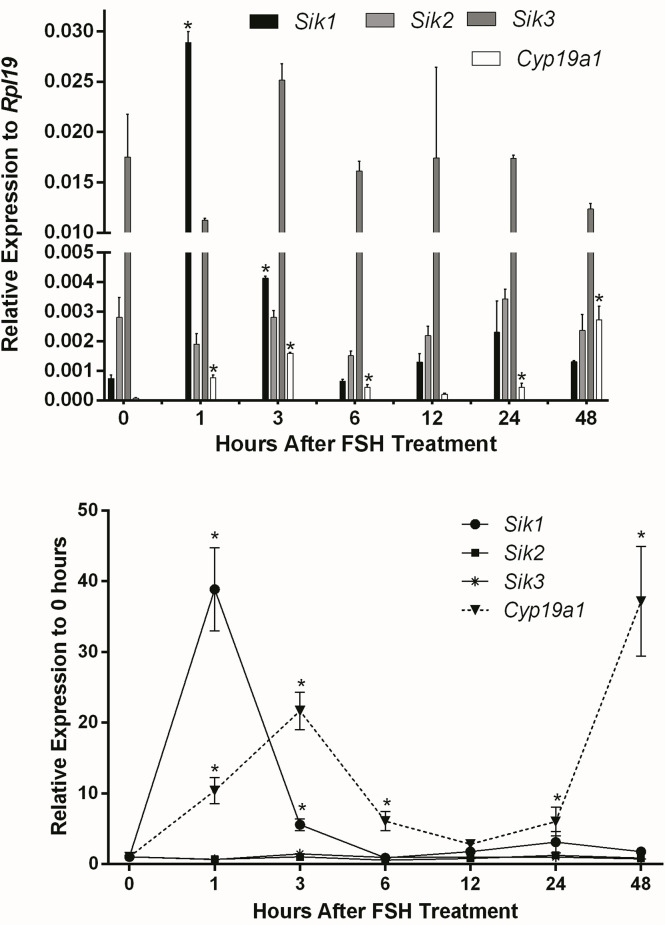
Effect of FSH on SIK expression. Rat GCs were treated with FSH (50 ng/ml). The mRNA levels for *Cyp19a1*, *Sik1*, *Sik2*, and *Sik3* were measured 1, 3, 6, 12, 24, and 48 h after the initiation of treatment. The expression of each gene is plotted as relative expression to *Rpl19* (Top) or relative to 0 h (Bottom). One-way ANOVA followed by Tukey. *p < 0.05, *n* = 3.

We previously showed *in vitro* and *in vivo* that SIK inhibition enhances FSH-induction of steroidogenic genes and estradiol production in human and rodent GCs (1). Therefore, we first determined whether FSH inhibits the expression of *Sik* in GCs. To test this, we treated rat GCs with FSH and measured *Sik1*, *Sik2*, and *Sik3* expression at 1, 3, 6, 12, 24, and 48 h after the initiation of the treatments. The expression of *Sik2* and *Sik3* remained consistent throughout the experiments (**Figure 1**). FSH induced a transient increase of *Sik1* after one hour of treatment. As expected, *Cyp19a1* was induced by FSH in a biphasic manner with a rapid increase at 1 and 3 h and a delayed increase at 24 and 48 h after the initiation of treatments. These results suggest that FSH effects on SIK do not correlate with its effects on the expression of steroidogenic genes. Thus, FSH does not decrease the expression of SIKs as we predicted.

### SIKs lessen FSH actions downstream of cAMP

*FSH receptor*: Next, we examined whether SIK inhibition increases the response of GCs to FSH by targeting the expression of the FSH receptor. GCs were treated with FSH in the presence or absence of 1 micromolar (10^-6^ M or µM) of HG-9-91-01 (HG). HG-9-91-01 is an effective SIK inhibitor, which has been shown to selectively target SIK proteins (26, 27). Cotreatment with FSH and HG or HG alone had no effects on FSH receptor (*Fshr*) expression (**Supplemental Figure 2**).

**Figure 2:**
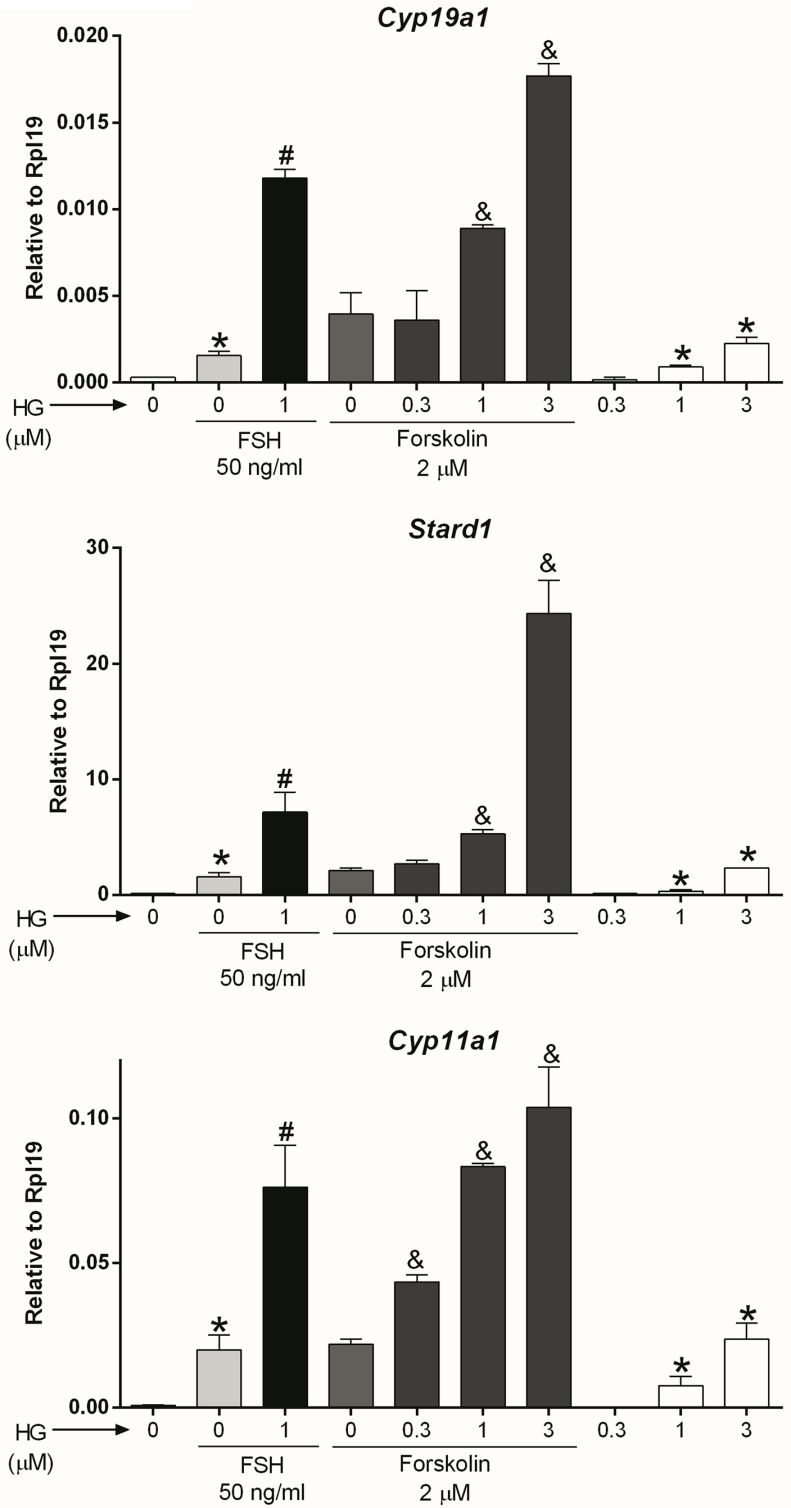
SIK inhibition enhances forskolin actions. Rat GCs were pretreated with vehicle or 0.3, 1, or 3 µM of HG for one hour; then, cells were treated with vehicle, FSH, or forskolin (an adenylate cyclase activator). *Cyp19a1*, *Stard1*, and *Cyp11a1* mRNA levels were determined 48 h after adding FSH or forskolin. One-way ANOVA followed by Tukey. *p < 0.05 vs. 0; #*p* < 0.01 vs. 0+FSH; &*p* < 0.01 vs. 0+forskolin; *n* = 4.

*Adenylyl cyclase (AC)*: After observing that SIK inhibition has no effect on FSH receptor expression, we hypothesized that it might affect signaling downstream of the FSH receptor. Since the FSH receptor activates the AC, we treated GCs with forskolin, a specific AC activator, in the presence or absence of 0.3, 1, or 3 micromolar (10^-3^ M, µM) of HG-9-91-01 (HG). These concentrations are based on our previous publications on GCs (1) and previous reports demonstrating HG high specificity to inhibit SIK activity (26, 27). Forskolin stimulated the expression of *Cyp19a1*, *Stard1*, and *Cyp11a1* (**Figure 2**). The stimulatory effect of forskolin was significantly enhanced in a concentration-dependent manner by the inhibition of SIK activity (**Figure 2**). Thus, *Cyp19a1* and *Stard1* induction by forskolin was enhanced by 1 and 3 µM HG. In contrast, *Cyp11a1* induction by forskolin was significantly augmented even by 0.3 µM (300 nM), the lowest concentration tested. In good agreement with our previous report (1), treatment with HG alone stimulated the expression of *Cyp19a1*, *Stard1*, and *Cyp11a1* in a concentration-dependent manner having significantly stimulatory effects at 1 and 3 µM.

**Figure 3:**
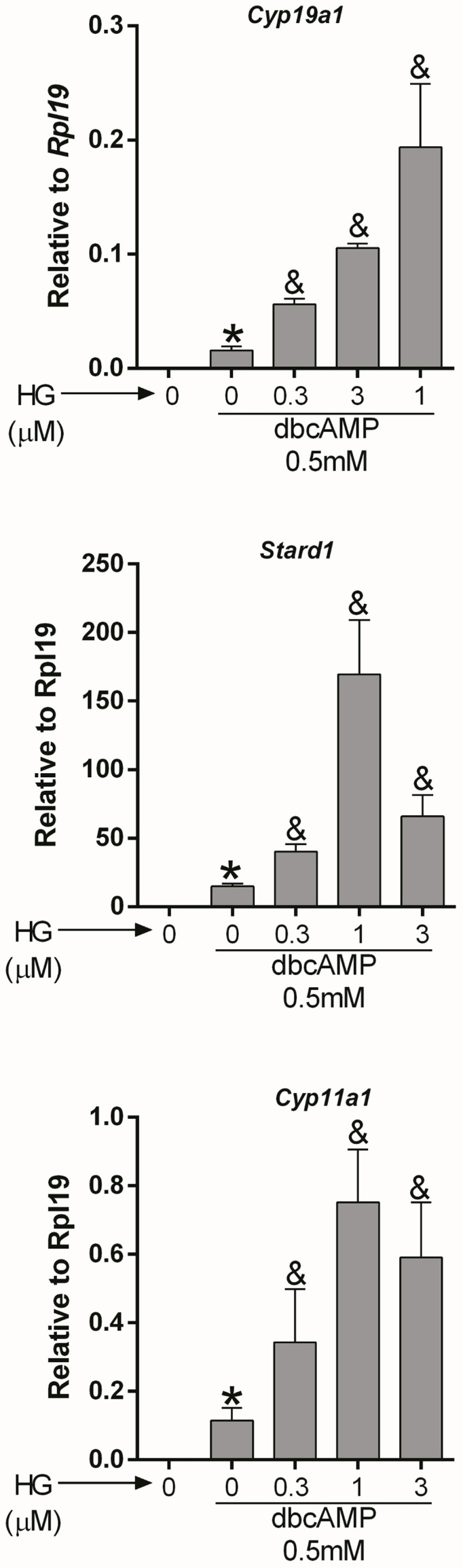
SIK inhibition enhances cAMP actions. Rat GCs were pretreated with vehicle or 0.3, 1, or 3 µM of HG for one hour; then, cells were treated with vehicle or dbcAMP (a cell-permeable analog of cAMP). *Cyp19a1*, *Stard1*, and *Cyp19a1* mRNA levels were determined 48 h after adding dbcAMP. One-way ANOVA followed by Tukey. *p < 0.05 vs. 0; &*p* < 0.01 vs. 0+dbcAMP, *n* = 3.

*Cyclic AMP*: AC activity increases intracellular cAMP; therefore, we next examined whether SIK inhibition also augments the stimulatory effect of cAMP on gene expression. For this purpose, we treated GCs with dibutyryl-cAMP (dbcAMP), a cell-permeable analog of cAMP, which alone induced *Cyp19a1*, *Stard1*, and *Cyp11a1* expression. As with FSH and forskolin, SIK inhibition augmented the stimulatory effect of dbcAMP on gene expression in a concentration-dependent manner (**Figure 3**). Thus, dbcAMP induction of *Cyp19a1*, *Stard1*, and *Cyp11a1* was significantly augmented by HG at 0.3, 1, and 3 µM. Although, in the case of *Stard1* and *Cyp11a1*, the enhancement of dbcAMP actions with 3 µM was not as strong as with 1 µM of HG.

cAMP effects are mediated mainly by two cAMP-binding proteins: the exchange protein directly activated by cAMP (EPAC) and PKA (28). Therefore, we next examined whether SIK inhibition modifies the effects of the pharmacological activation of EPAC with 8-(4-chlorophenylthio) adenosine 3’5’-cAMP (8CPT). 8CPT activates EPAC but not PKA (29). EPAC activation did not induce *Cyp19a1*, *Stard1*, and *Cyp11a1* expression, whereas SIK inhibition alone induced these genes. The combination of HG and 8CPT treatment did not stimulate gene expression beyond the stimulation levels of HG treatment alone (**Supplemental Figure 3**).

### SIK inhibition enhances constitutively active PKA effects

**Figure 4:**
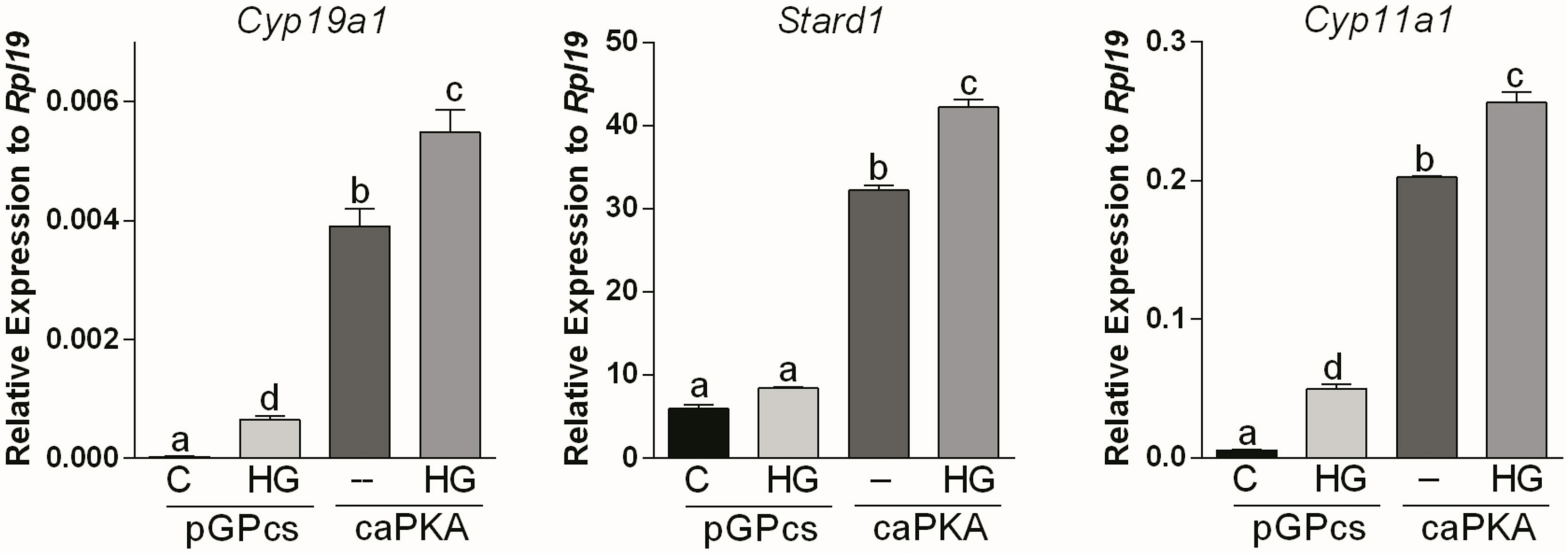
SIK inhibition enhances constitutively active PKA stimulation of gene expression. Rat GCs were infected 2 h after plating with lentivirus carrying an empty plasmid (pGPcs) or caPKA. 24 h later, cells were treated with vehicle or HG (0.5 µM). *Cyp19a1*, *Stard1*, and *Cyp11a1* mRNA levels were determined 48 h after adding HG. One-way ANOVA followed by Tukey. Columns labeled with different letters differ significantly a-d and b-c, *p* < 0.05; a-c, a-b, *p* < 0.01, *n* = 4.

Next, we used a lentivirus to express constitutively active PKA (caPKA) protein, which carries His87Gln and Trp196Arg mutations rendering it insensitive to the regulatory units (20). We first determined if caPKA increases the activity of a cAMP response element reporter (CRE-Luc). As shown in **Supplemental Figure 1**, overexpression of caPKA significantly stimulated the activity of the CRE-Luc reporter. Next, we infected GCs with lentivirus carrying an empty plasmid (pGPcs) or caPKA. Overexpression of caPKA was sufficient to stimulate *Cyp19a1*, *Stard1*, and *Cyp11a1* expression an effect that was significantly enhanced by SIK inhibition (**Figure 4**).

### Effect of SIK inhibition on PKA downstream targets

**Figure 5:**
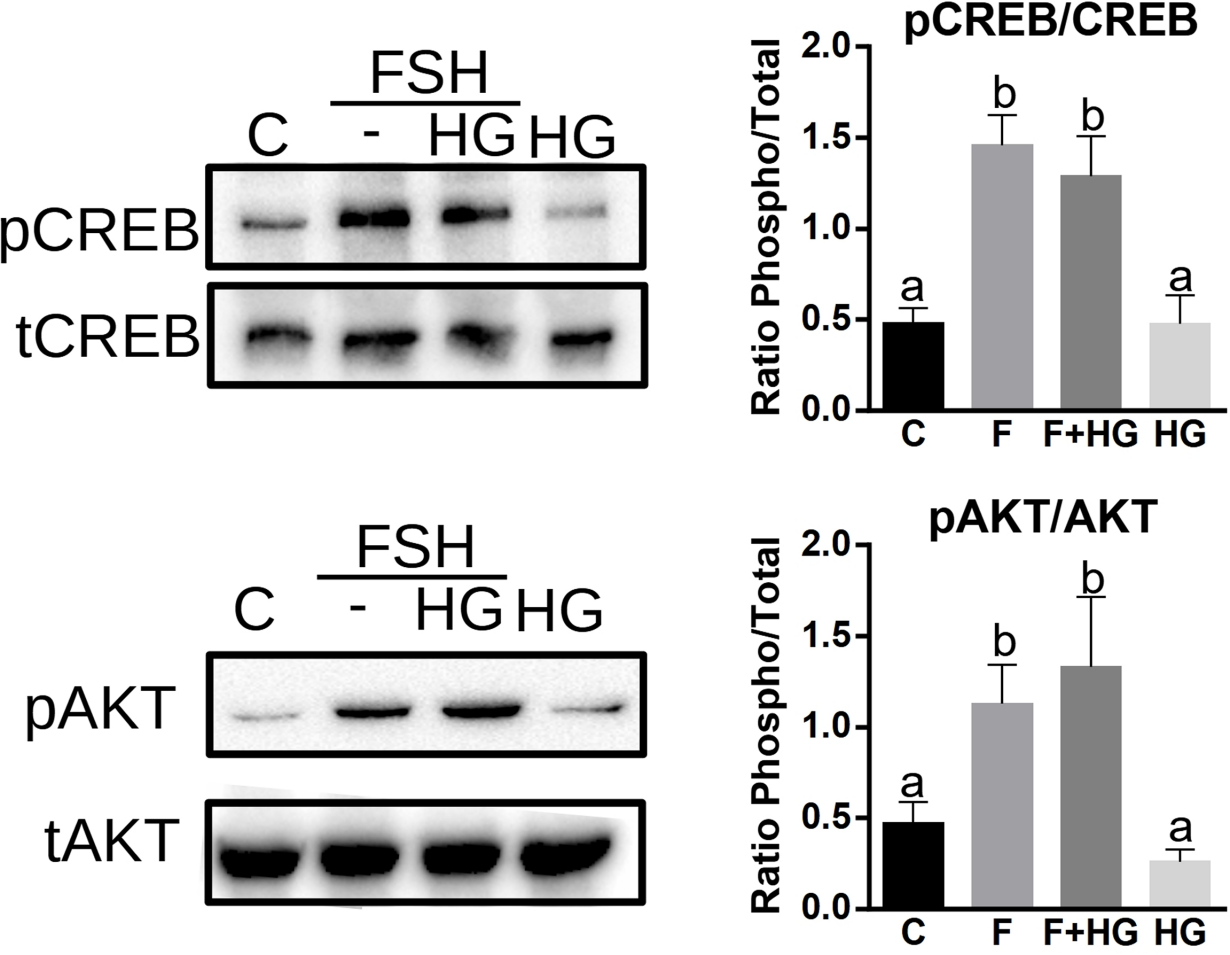
SIK inhibition does not increase CREB or AKT phosphorylation. Cultured rat GCs were pretreated with HG (0.5 µM) for one hour and then stimulated with FSH for one hour. Whole-cell lysates were used for total CREB, phospho-S133-CREB, total AKT, and phospho-S473-AKT determination by Western blotting. The experiment was repeated 3 times, a representative blot is shown. For all experiments, bands were quantified and the ratio of phospho to total protein was calculated, the average ± SEM is plotted on the right. One-way ANOVA followed by Tukey. Columns labeled with different letters differ significantly a-b p < 0.05, *n* = 3.

PKA activates AKT and CREB in GCs (19, 30, 31); we next sought to determine if SIK activity impacts the activation of these proteins. GCs were treated with FSH in the presence or absence of HG for one hour, and then whole-cell lysates were used for total and phospho AKT and CREB determination using Western blotting. As expected, FSH significantly increased AKT and CREB phosphorylation. The inhibition of SIK activity with HG did not modify the stimulatory effect of FSH on AKT or CREB phosphorylation. SIK inhibition alone had no effects on AKT or CREB phosphorylation (**Figure 5**).

### SIK inhibition increases the activity of a CRE reporter

**Figure 6:**
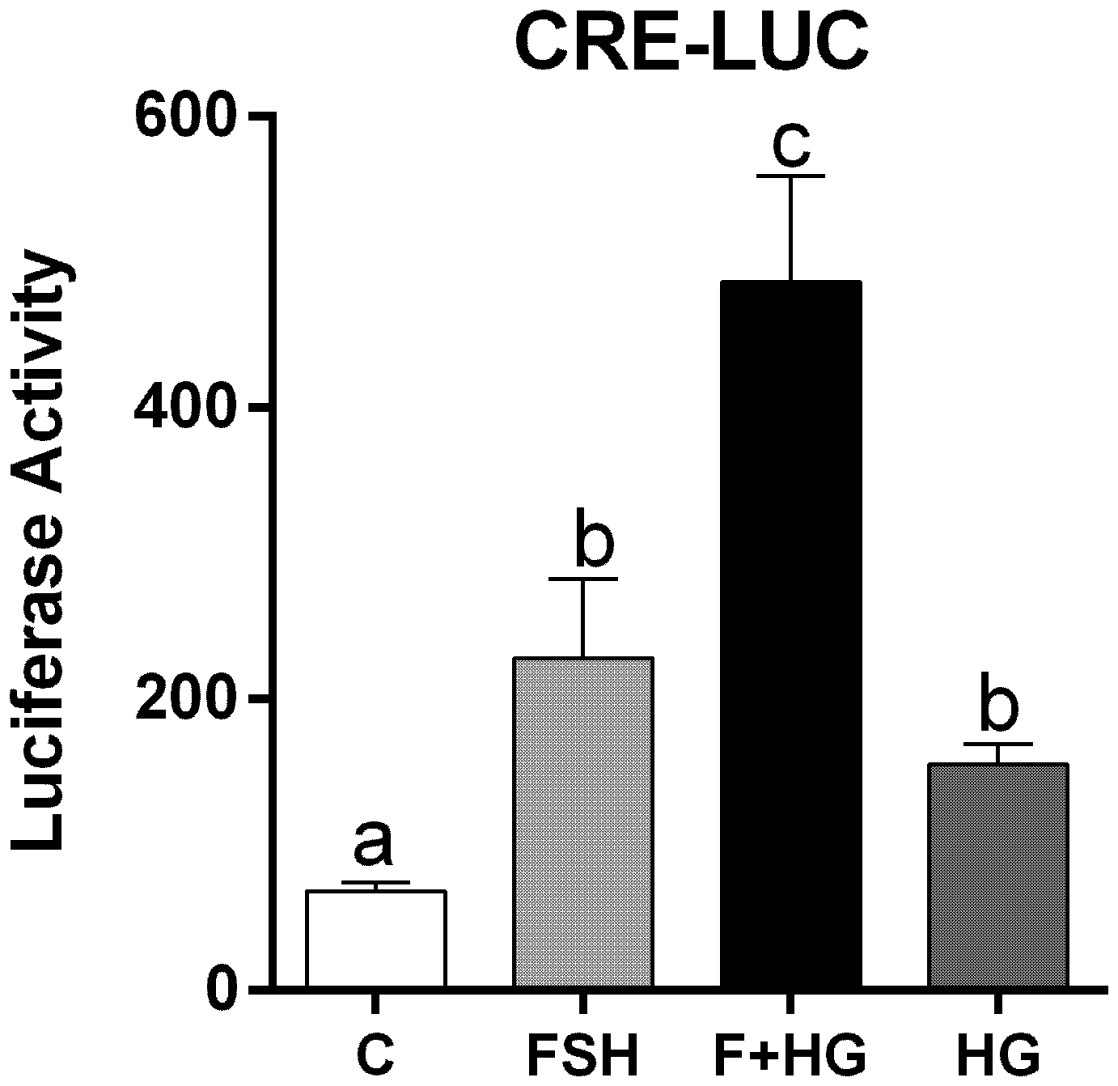
Effect of FSH on cAMP response element activation. Rat GCs were infected 2 h after plating with lentivirus carrying an empty plasmid or the pCRE-LUC reporter. 24 h later, cells were treated with FSH (50 ng/ml) in the presence or absence of HG (0.5 µM). Luciferase activity was quantified 48 h after adding FSH and HG. One-way ANOVA followed by Tukey. Columns labeled with different letters differ significantly a-b and b-c, *p* < 0.05; a-c *p* < 0.01, *n* = 3.

Since inhibition of SIK activity did not augment CREB phosphorylation, we next investigated the effect of SIK inhibition on CREB activity. For this purpose, we created a cAMP response element reporter (CRE-Luc, see materials and methods). As expected, CRE-Luc activity increased significantly after treatment with FSH. HG treatment enhanced FSH-induced CRE-Luc activity and alone increased CRE-Luc activity significantly (**Figure 6**).

**Figure 7:**
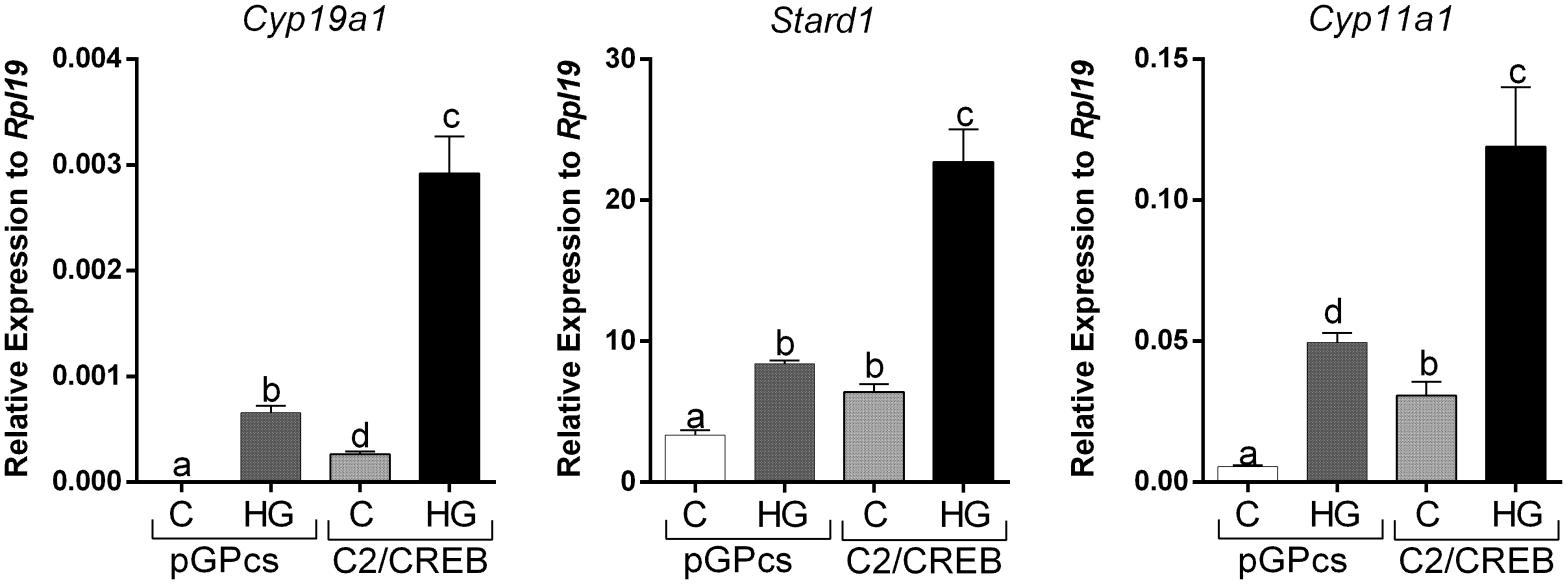
SIK inhibition enhances C2/CREB stimulation of gene expression. Rat GCs were infected 2 h after plating with lentivirus carrying an empty plasmid (pGPcs) or C2/CREB. 24 h later, cells were treated with vehicle or HG (0.5 µM). *Cyp19a1*, *Stard1*, and *Cyp11a1* mRNA levels were determined 48 h after adding HG. One-way ANOVA followed by Tukey. Columns labeled with different letters differ significantly a-b, a-d, and b-d, p < 0.05; a-c, and b-c p < 0.01, *n* = 3.

To outline possible mechanisms by which SIKs might regulate CRE-Luc activity without affecting CREB phosphorylation, we tested the impact of SIK inhibition on the stimulation of gene expression by C2/CREB. C2/CREB is a fusion protein that activates CREB-responsive genes in the absence of cAMP or PKA activation (21). Initial experiments showed that overexpression of C2/CREB increases the activity of the CRE-Luc reporter (**Supplemental Figure 1**). Then, we infected GCs with lentivirus carrying an empty plasmid (pGPcs) or C2/CREB. C2/CREB overexpression induced *Cyp19a1*, *Stard1*, and *Cyp11a1* expression. Inhibition of SIK activity in cells overexpressing C2/CREB augmented *Cyp19a1*, *Stard1*, and *Cyp11a1* expression to levels that were significantly higher than those observed with C2/CREB alone (**Figure 7**).

### SIK inhibition regulates the nuclear translocation of CREB coactivators

**Figure 8:**
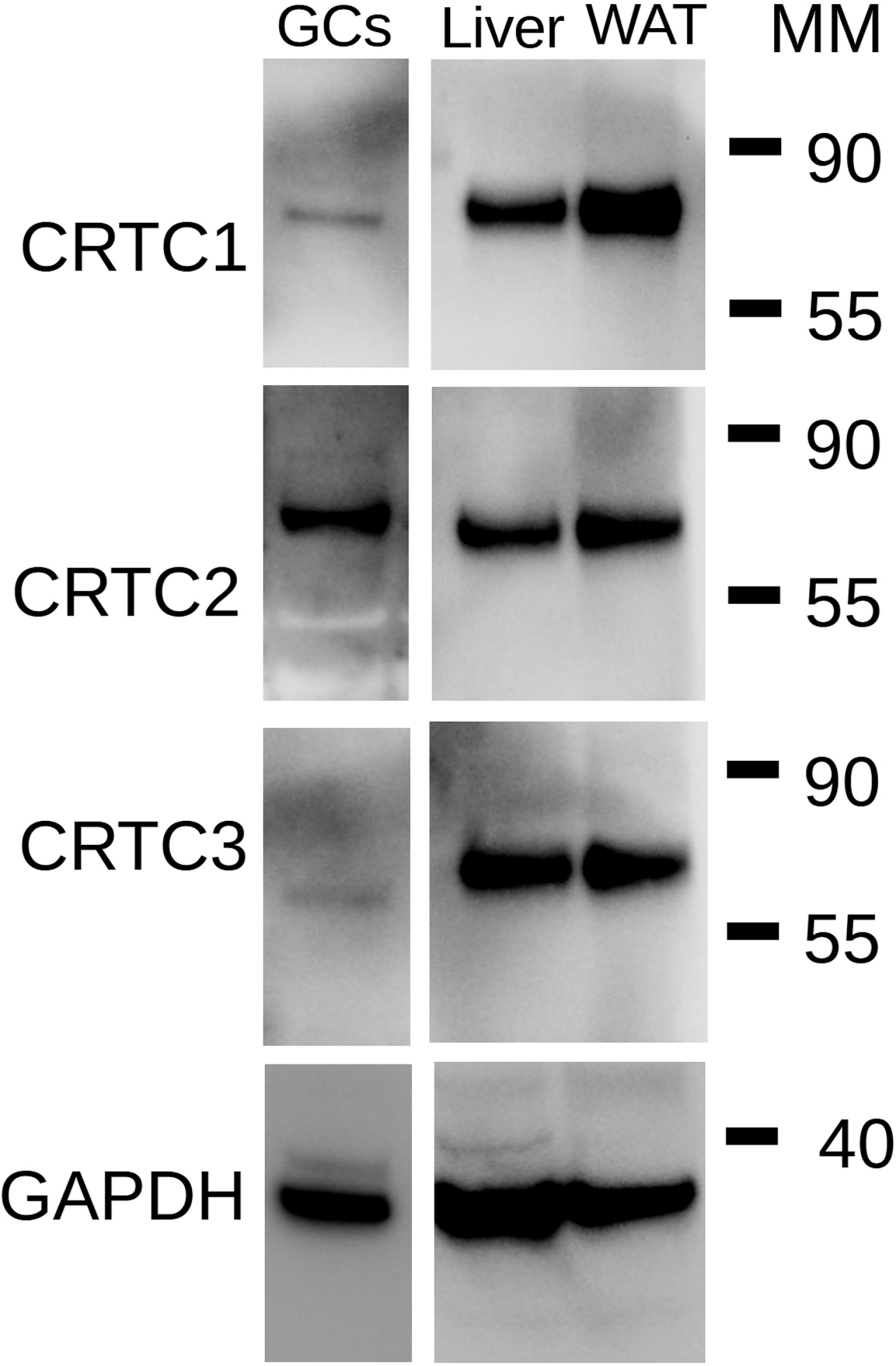
Expression of CRTC isoforms in GCs. Total protein extracts from rat GCs were used for Western blot analysis for CRTC1, CRTC2, CRTC3, and GAPDH (loading control). Extract of rat liver and rat white adipose tissue (WAT) were used as positive controls. MM: Molecular Marker weight in kDa. Representative blot of two individual determinations.

The lack of effect of SIK inhibition on CREB phosphorylation and its capacity to enhance the induction of CRE-Luc activity by FSH or the stimulation of steroidogenic gene expression by C2/CREB led to the hypothesis that SIK inhibition regulates the activity of CREB coactivators. SIKs are known to regulate CREB-regulated transcriptional coactivators (CRTCs) (22). As this family of factors contains three members (CRTC1, CRTC2, and CRTC3), we first examined which isoform is expressed in rat GCs. As shown in **Figure 8**, CRTC2 is the main isoform expressed in the GCs. GCs express low levels of CRTC1 but lack CRTC3. Protein extracts from liver and white adipose tissue were used as positive controls for the expression of CRTC isoforms.

**Figure 9:**
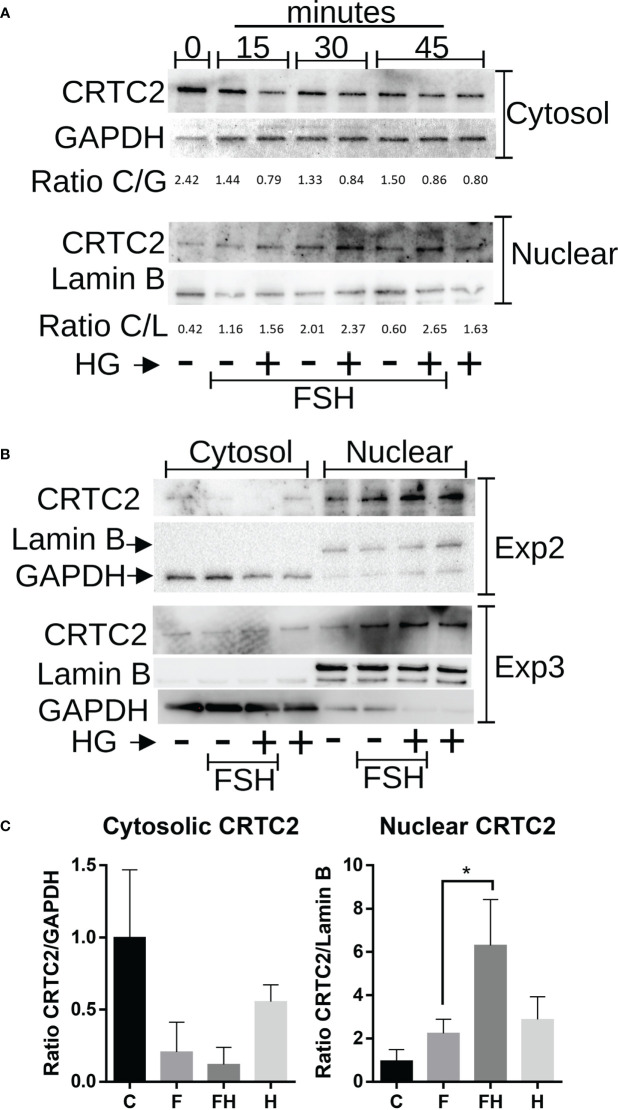
Effect of FSH and SIK inhibition on the subcellular localization of CRTC2. **(A)** Rat GCs were treated with FSH (50 ng/ml) in the presence or absence of HG (0.5 µM) for 15, 30, or 45 minutes or left untreated. **(B)** Rat GCs were treated with FSH in the presence or absence of the SIK inhibitor HG for 45 minutes. For A and B, Nuclear and cytosolic extracts were prepared and blotted for CRTC2, GAPDH (cytosolic marker), or Lamin B (nuclear marker). The bands were quantified using Image J software, and the ratio between CRTC2 and the corresponding subcellular marker was calculated. **(C)** Cytosolic and Nuclear CRTC2 content as a ratio to the corresponding subcellular marker is plotted. *p <0.05, n = 3.

CRTCs activity is mainly regulated by their translocation to the nucleus, where they contribute to activating gene expression (22). Therefore, we next examined the effects of FSH and SIK inhibition on the subcellular localization of CRTC2. GCs were treated with FSH in the presence or absence of HG (1µM) for 0, 15, 30, or 45 minutes. Following this treatment, cytosolic and nuclear protein fractions were prepared. The content of CRTC2 in the nuclear fraction increased with FSH treatment. Combined treatment with FSH and the SIK inhibitor increased the expression of CRTC2 in the nuclear fraction, especially after 30 and 45 minutes of treatment (**Figure 9A**). Based on these findings, we repeated the experiment two more times using 45 minutes of treatment with FSH in the presence or absence of HG. As shown in **Figure 9B**, FSH increased CRTC2 content in the nucleus while cotreatment with HG enhanced FSH effects. Finally, we quantified cytosolic and nuclear CRTC2 levels at 45 minutes and expressed them as a ratio to GAPDH or Lamin B, which were used as loading controls for the cytosolic and nuclear fractions, respectively. The analysis demonstrated that treatment with FSH and HG significantly increases CRTC2 expression in the nuclear fractions compared to control and FSH alone (**Figure 9C**).

**Figure 10:**
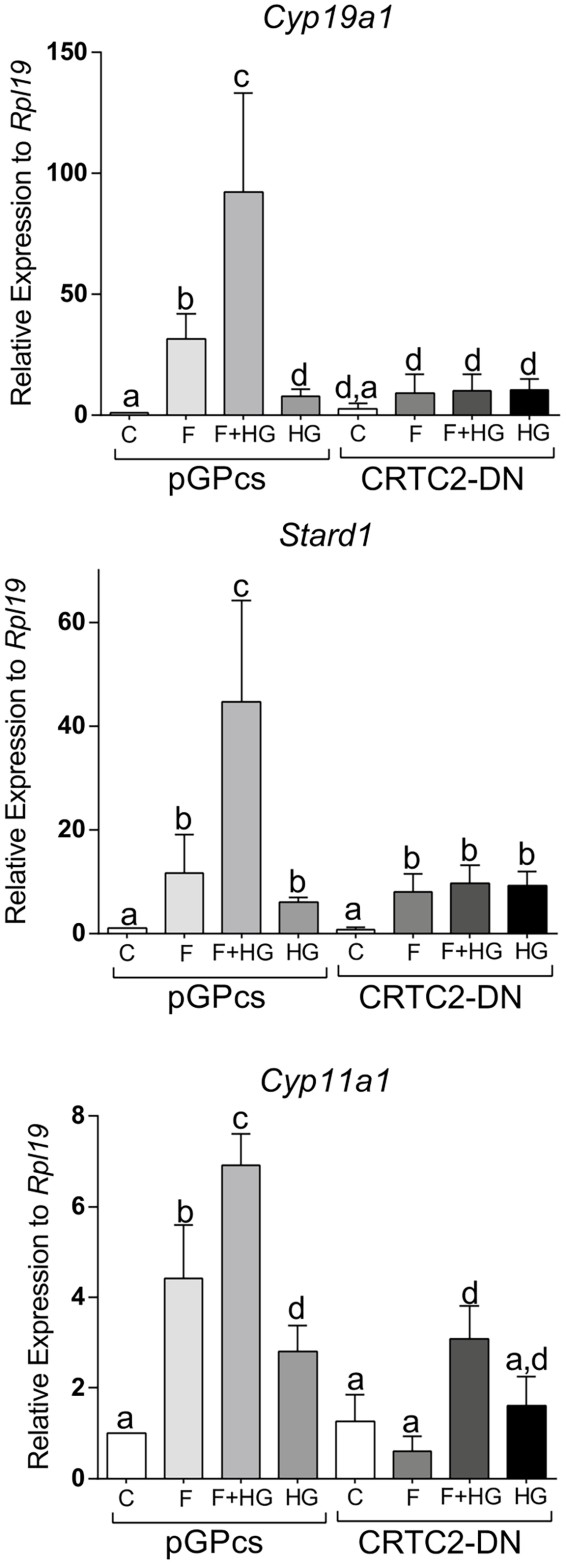
Dominant negative CRTC2 inhibits FSH and SIK inhibition effects. Rat GCs were infected 2 h after plating with lentivirus carrying an empty plasmid (pGPcs) or CRTC2-DN. 24 h later, cells were treated with FSH (50 ng/ml) in the presence of vehicle or HG (0.5 µM). *Cyp19a1*, *Stard1*, and *Cyp11a1* mRNA levels were determined 48 h after adding FSH. One-way ANOVA followed by Tukey. Columns labeled with different letters differ significantly a-b, a-d, and b-c, *p* < 0.05; a-c *p* < 0.01, *n* = 3.

Since SIK inhibition enhances the translocation of CRTC2 to the nucleus, we next examined the effect of dominant negative CRTC2 (CRTC2-DN), which has been shown to block the effects of all CRTCs (23, 24). Expression of the CRTC2-DN blocked the induction of *Cyp19a1*, *Stard1*, and *Cyp11a1* expression by FSH or by the combination of FSH plus HG (**Figure 10**).

## Discussion

We recently described the expression of SIK isoforms in the ovary and GCs and showed that inhibition of SIK activity enhances FSH actions *in vitro* and *in vivo* (1). Thus, we showed that SIK inhibition enhances FSH induction of steroidogenic gene expression and estradiol production in human and rodent GCs (1). In addition, analysis of SIK knockout mice demonstrated that SIKs are critical regulators of female fertility. The main objective of this report was to determine the intracellular mechanisms by which SIK inhibition enhances FSH actions in the GCs.

Our previous report demonstrated that inhibition of SIK activity is enough to mimic FSH actions (1). Therefore, we initially hypothesized that FSH might, at least in part, stimulate the expression of *Cyp19a1*, *Stard1*, and *Cyp11a1* and the production of estradiol by decreasing SIKs expression. However, the results show that FSH does not inhibit SIKs expression up to 48 h after treatment, which is the peak of FSH-induced *Cyp19a1* expression. We also showed that SIK inhibition has no effects on the expression of the FSH receptor, suggesting that SIKs act downstream of the FSH receptor. Instead of a decrease in SIKs expression as expected, we found that FSH transiently stimulates the expression of SIK1. SIK1 is also stimulated by adrenocorticotropic hormone in the adrenal glands (5). The transient induction of SIK1 may contribute to the control of FSH actions by providing negative feedback on FSH receptor signaling. However, the role of SIK1 stimulation by FSH in GCs remains to be investigated.

Our findings suggest that SIKs target signaling molecules downstream of cAMP, as evidenced by the ability of SIK inhibition to enhance *Cyp19a1*, *Stard1*, and *Cyp11a1* expression induced by forskolin and dbcAMP. Forskolin causes direct activation of adenylyl cyclase activity, stimulating the production of cAMP, whereas dbcAMP mimics the action of endogenous cAMP. Therefore, the robust enhancement of forskolin- and dbcAMP-induced stimulation of gene expression by SIK inhibition indicates that SIK activity controls targets downstream of cAMP. Moreover, our findings demonstrate that of the two cAMP targets, PKA and EPAC, SIK activity appears to regulate only PKA downstream signaling. EPAC has no effects on the stimulatory effects of SIK inhibition on gene expression. However, SIK inhibition augments the stimulatory effects of caPKA on *Cyp19a1*, *Stard1*, and *Cyp11a1* expression. Thus, our findings show that SIKs act downstream of PKA.

Strikingly, although SIK inhibition potentiates PKA actions in the GCs, inhibiting SIK activity has no impact on CREB phosphorylation, a primary target of FSH and PKA in GCs (32, 33). CREB is rapidly and transiently phosphorylated by FSH (12, 34), whereas overexpression of a non-phosphorylatable mutant of CREB in primary cultures of rat GCs decreases estradiol production induced by FSH and adversely affects GC survival (33). This evidence suggests that CREB activation is required for normal GC differentiation. Surprisingly, SIK inhibition increases the activity of a CRE reporter and the expression of several CREB-dependent genes such as *Cyp19a1*, *Stard1*, and *Cyp11a1*. Moreover, we demonstrated that SIK inhibition enhances the stimulation of gene expression induced by C2/CREB, a CREB fusion protein that stimulates CRE-responsive genes in the absence of cAMP. This evidence demonstrates that SIK does not directly target CREB but factors that might enhance its activity.

Previous reports have demonstrated that SIKs are potent inhibitors of CRTCs (26, 35–39). CRTC movement between the nucleus and the cytoplasm is regulated by phosphorylation (6). In particular, CRTC phosphorylation by SIK results in nuclear exclusion (40, 41). Under basal conditions, CRTCs are sequestered in the cytoplasm. However, activation of specific pathways causes CRTC nuclear translocation and binding to CREB. The binding of CRTCs to CREB also leads to increased CREB occupancy over cognate binding sites (42). Therefore, CREB activity is not only regulated by phosphorylation but also by the translocation of CRTC2 to the nucleus. Our findings show for the first time that rat GCs express mainly CRTC2 and low levels of CRTC1. Moreover, we observed that treatment with FSH leads to the translocation of CRTC2 to the nucleus, an effect significantly augmented by the simultaneous inhibition of SIK activity. Because FSH induction of steroidogenic genes is decreased by the overexpression of CRTC2-DN, we propose that SIKs blunt FSH signaling in the GCs by maintaining CRTC2 in the cytoplasm.

It is known that CRTCs activate CREB independent of S133 phosphorylation (23, 41) and that CRTC nuclear translocation is necessary and sufficient for CRE activation (23). We have previously reported that CREB localizes to the nucleus of GCs and binds to the *Cyp19a1* promoter even in the absence of FSH (43). Here, we show that SIK inhibition is enough to stimulate CRTC2 nuclear translocation in GCs, which coincides with increased steroidogenic gene expression and CRE-Luc activity. These findings suggest that the nuclear translocation of CRTC2 may also explain the stimulatory effect of SIK inhibition on *Cyp19a1*, *Stard1*, and *Cyp11a1* in the absence of FSH. Thus, CRTC2 movement to the nucleus is enough to stimulate the GC differentiation program, most probably by activating CREB-occupied promoters. However, from our findings, it is also clear that full activation of the GC differentiation program is only reached in the presence of FSH. Whether CRTC2 regulates the activity of other transcription factors in addition to CREB in GCs remains to be investigated. In addition, because SIK inhibition alone is sufficient to stimulate the expression of markers of GC differentiation, it is possible to postulate that SIKs are highly active in undifferentiated GCs. Therefore, we postulate that SIK activity is inhibited by FSH. However, the mechanisms involved have not yet been explored.

In addition to cAMP, we have previously demonstrated that the AKT signaling pathway is essential for FSH-induced preovulatory GC differentiation (19). FSH stimulates AKT signaling (44); subsequently, a dominant-negative mutant of AKT blocks FSH-stimulated estrogen production (45). Despite the importance of AKT in FSH-induced GC differentiation, SIK inhibition has no effects on the phosphorylation and activation of AKT by FSH. Noteworthy, SIK activity is increased in the presence of active glycogen synthase kinase-3β (GSK3β) (9). FSH strongly and rapidly phosphorylates GSK3β in ovarian GCs (44). GSK3β activity is decreased by phosphorylation, particularly by AKT (46, 47). Therefore, we propose that in GCs, CRTC2 activity may also be increased by FSH *via* the activation of AKT, which in turn causes GSK3β and SIKs inactivation. Further experiments are needed to test this hypothesis.

In summary, our findings outline the intracellular signaling pathway downstream of the FSH receptor that is affected by SIK activity. We show that SIK inhibition enhances the actions of all the components of the FSH signaling transduction pathway, including adenylyl cyclase, cAMP, PKA, and CREB. Moreover, we show that FSH and SIKs interact to control the expression of CRTC2 in the nucleus of GCs. We propose that in GCs, SIK inhibition increases the recruitment of CRTC2 to the promoter of steroidogenic genes. Further experiments are needed to determine the involvement of CRTC2 in regulating CREB activity and whether SIK activity targets additional PKA substrates in ovarian GCs.

## Data availability statement

The original contributions presented in the study are included in the article/**Supplementary Material**. Further inquiries can be directed to the corresponding author.

## Ethics statement

The animal study was reviewed and approved by The Institutional Animal Care and Use Committee at the University of Illinois at Chicago.

## Author contributions

Conceptualization: MA and CS, Methodology: MA and MR, Formal analysis and investigation: MA, MR, and CS, Original draft preparation: MA and MR, Writing, review, and editing: MA, MR, and CS, Project administration and funding acquisition: CS. All authors contributed to the article and approved the submitted version.

## Funding

The authors thank the National Institute of Health (NIH) for the financial support grant R01HD097202 to CS.

## Acknowledgments

The authors thank Dr. Esfandyari for her technical support.

## Conflict of interest

The authors declare that the research was conducted in the absence of any commercial or financial relationships that could be construed as a potential conflict of interest.

## Publisher’s note

All claims expressed in this article are solely those of the authors and do not necessarily represent those of their affiliated organizations, or those of the publisher, the editors and the reviewers. Any product that may be evaluated in this article, or claim that may be made by its manufacturer, is not guaranteed or endorsed by the publisher.

## Supplementary material

Supplementary Figure 1 caPKA and c2/CREB capacity to activate a CRE-reporter. HEK293 cells were transfected with pCRE-LUC plus empty vector, caPKA, or C2/CREB. Luciferase activity was determined 48 h after transfection. ** p<0.01 vs. empty, *n* = 3.

Supplementary Figure 2 Effect of SIK inhibition on FSH receptor expression. Rat GCs were treated with FSH in the presence or absence of HG. FSH receptor mRNA levels were measured 48 h after the initiation of treatments. No significant differences were found. One-way ANOVA followed by Tukey, *n* = 3.

Supplementary Figure 3 Activation of EPAC does not affect SIK inhibition effects. Rat GCs were pretreated with vehicle or HG (0.5 µM) for one hour; then, cells were treated with vehicle, FSH, or 10 µM 8-CPT-cAMP (8CPT), an EPAC activator. Aromatase, StAR, and P450scc mRNA levels were determined 48 h after adding FSH or 8CPT. One-way ANOVA followed by Tukey. *p < 0.05, **p < 0.01, n = 3.
