# Supplementary figures and images for: Mechanism of negative modulation of FSH signaling by salt-inducible kinases in rat granulosa cells
Source: Front Endocrinol (Lausanne). 2022 Sep 29;13:1026358. doi: 10.3389/fendo.2022.1026358 (PMC9556844; doi:10.3389/fendo.2022.1026358)

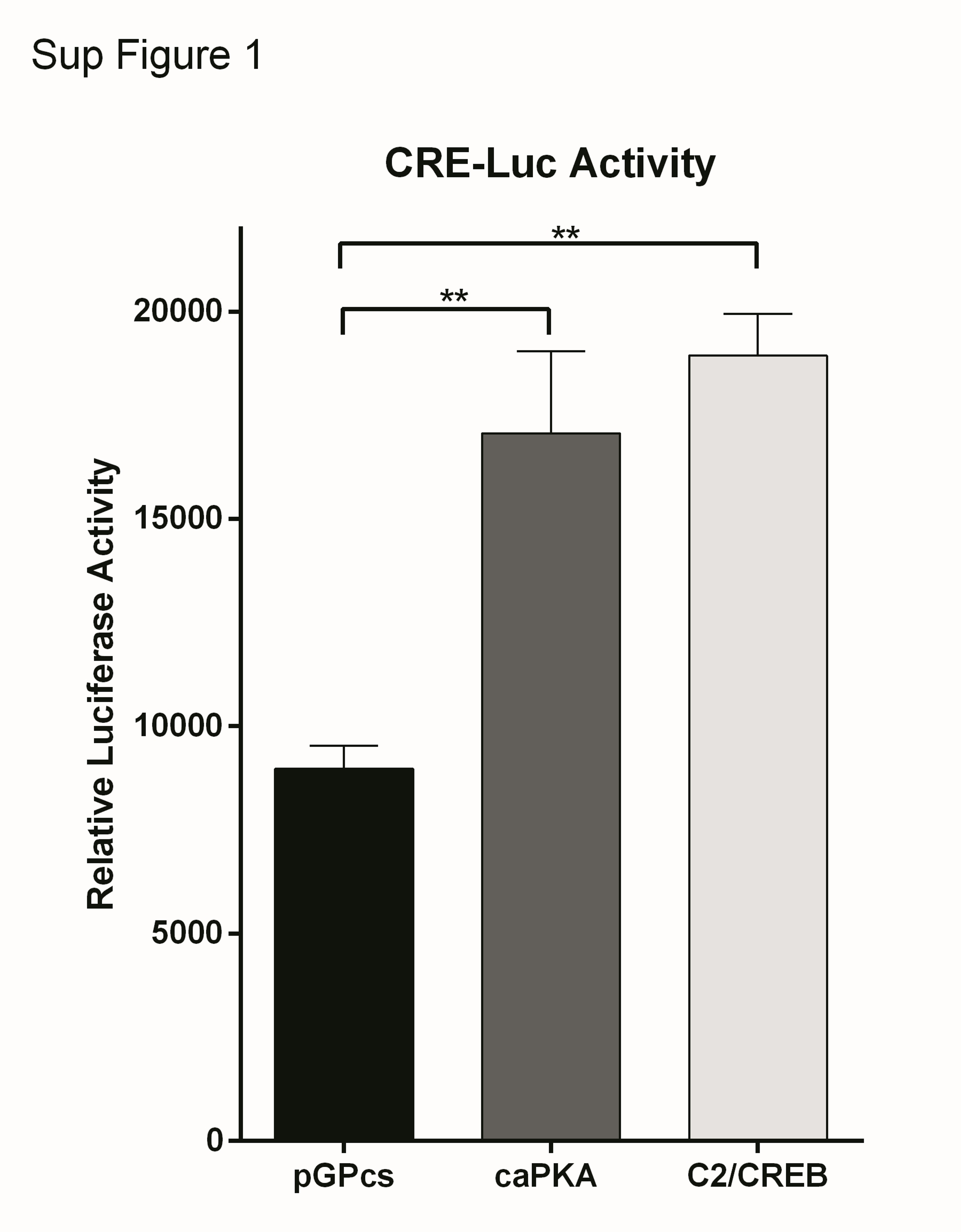

Supplement: Supplementary Figure 1 — caPKA and c2/CREB capacity to activate a CRE-reporter. HEK293 cells were transfected with pCRE-LUC plus empty vector, caPKA, or C2/CREB. Luciferase activity was determined 48 h after transfection. ** p<0.01 vs. empty, n = 3. [file Image_1.jpeg]

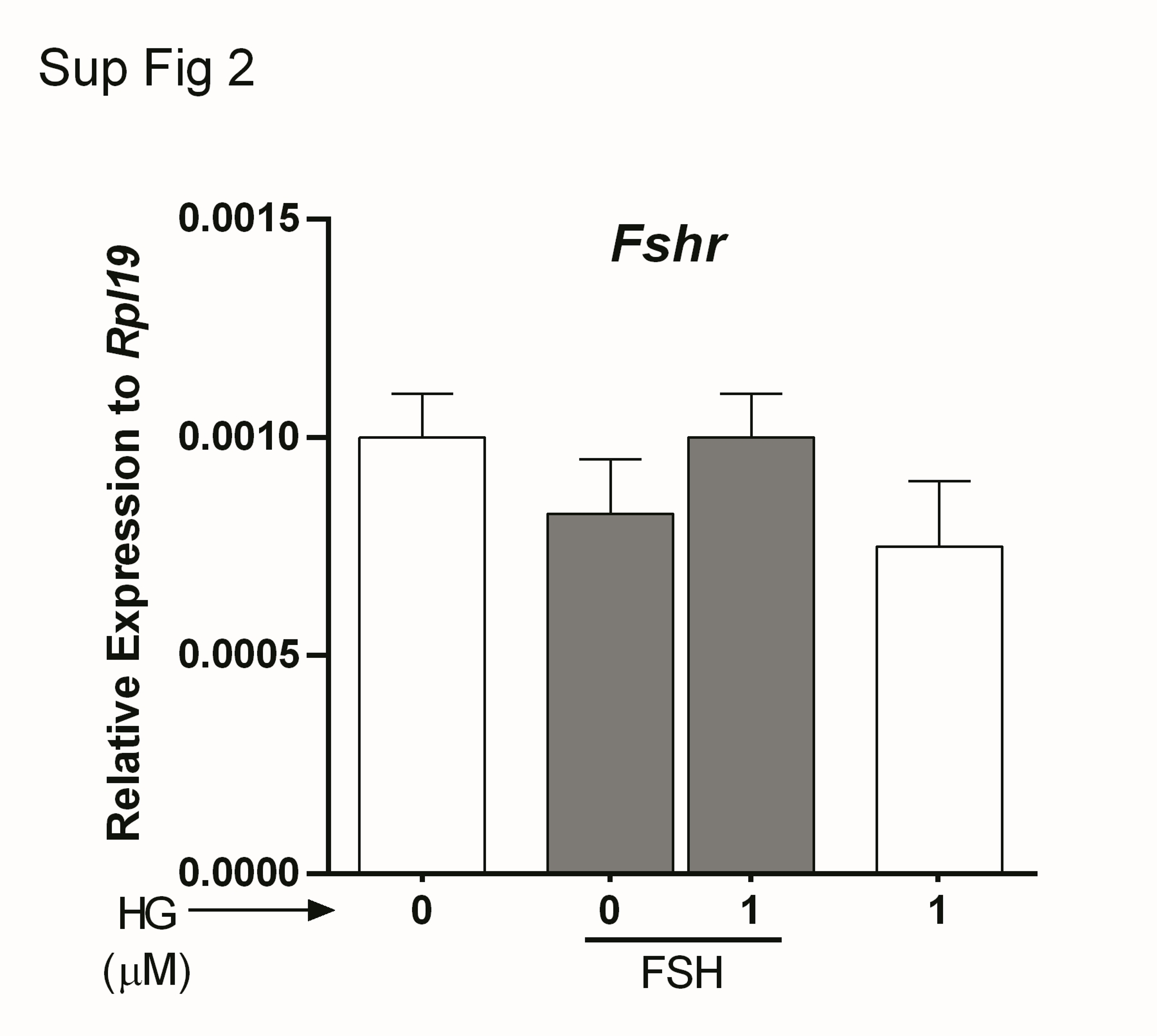

Supplement: Supplementary Figure 2 — Effect of SIK inhibition on FSH receptor expression. Rat GCs were treated with FSH in the presence or absence of HG. FSH receptor mRNA levels were measured 48 h after the initiation of treatments. No significant differences were found. One-way ANOVA followed by Tukey, n = 3. [file Image_2.jpeg]

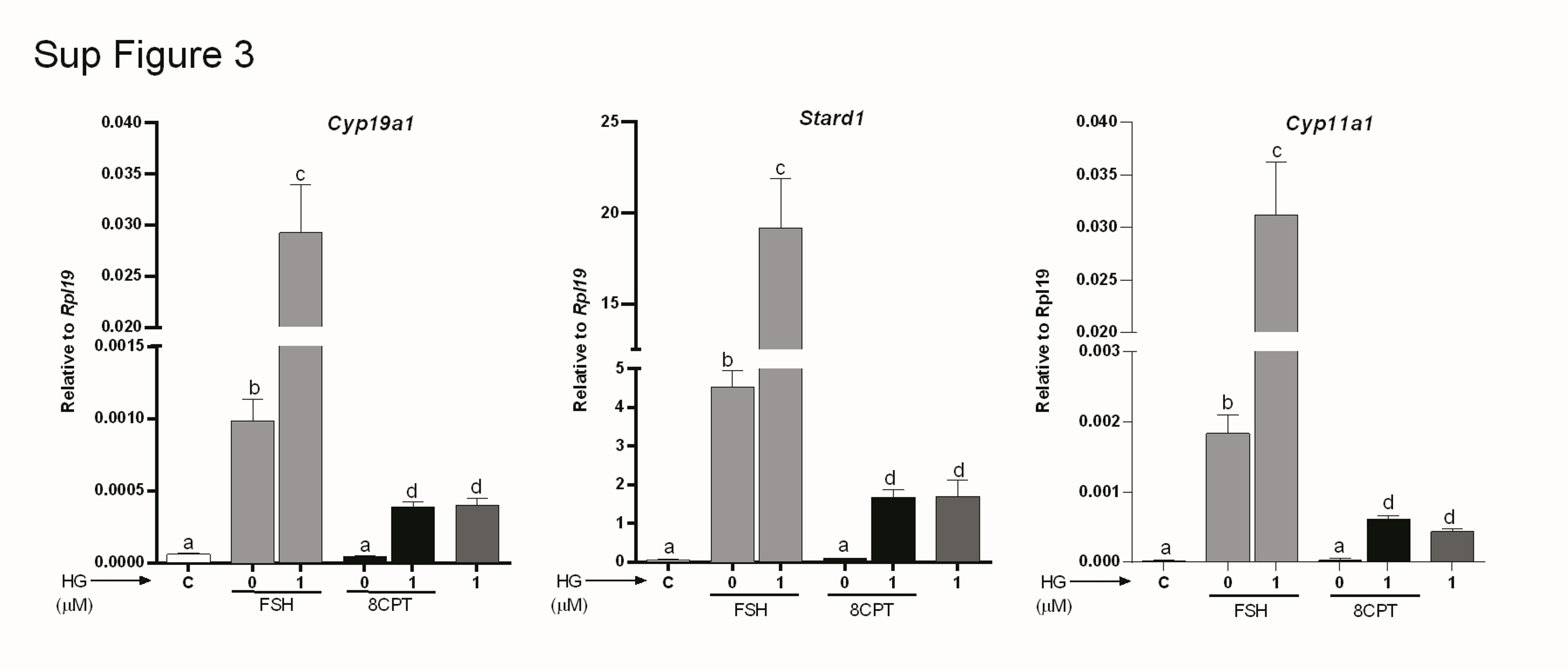

Supplement: Supplementary Figure 3 — Activation of EPAC does not affect SIK inhibition effects. Rat GCs were pretreated with vehicle or HG (0.5 µM) for one hour; then, cells were treated with vehicle, FSH, or 10 µM 8-CPT-cAMP (8CPT), an EPAC activator. Aromatase, StAR, and P450scc mRNA levels were determined 48 h after adding FSH or 8CPT. One-way ANOVA followed by Tukey. *p < 0.05, **p < 0.01, n = 3. [file Image_3.jpeg]
